# The Dual Protection of a Micro Land Snail against a Micro Predatory Snail

**DOI:** 10.1371/journal.pone.0054123

**Published:** 2013-01-09

**Authors:** Shinichiro Wada, Satoshi Chiba

**Affiliations:** Graduate School of Life Sciences, Tohoku University, Aobayama, Sendai, Miyagi, Japan; Australian Museum, Australia

## Abstract

Defense against a single predatory attack strategy may best be achieved not by a single trait but by a combination of different traits. We tested this hypothesis experimentally by examining the unique shell traits (the protruded aperture and the denticles within the aperture) of the micro land snail *Bensonella plicidens*. We artificially altered shell characteristics by removing the denticles and/or cutting the protruded aperture. These snails were offered to the carnivorous micro land snail *Indoennea bicolor*, which preys on the snails by gaining entry to their shell. *B. plicidens* exhibited the best defence when both of the traits studied were present; the defensive ability of *B. plicidens* decreased if either trait was removed and was further reduced if both traits were removed. These results suggest that a combination of different traits provides more effective defence against attack by the predator than either single trait by itself.

## Introduction

Predation is an important cause of evolutionary change in many prey taxa, and a hard shell or carapace is one of the most common defensive traits in several animal groups. Some species have stinging armour that is an effective defence against predation, e.g., the threespine stickleback [1]. It has also been suggested that mimicry and camouflage are effective traits to escape the attack of predators, e.g., many insect [2], dragon lizard [3] and octopus [4] taxa. Evolutionary change against predation is not limited to morphology. Many types of plants produce chemicals against herbivores [5], and the North American newt *Taricha granulosa* produces a high level of poison [6]. The evolution of such defensive traits would have been promoted by predation and coevolutionarily developed by the prey-predator interaction.

Although the function of single traits has been the focus of the majority of studies addressing the topic of defence by prey species against attack by predators, most prey species develop several different defensive traits. For example, most octopus species have at least two defensive strategies: camouflage and releasing the contents of the ink sac. Armadillos (Cingulata) also have two defensive traits a leathery armour shell and the ability to roll up the body; although each trait is insufficient to protect thebody against predation, armadillos frequently implement a high-performance defence through a combination of these defensive traits [7]. The effects of predation on individual defensive traits are well studied, yet relatively little is known about the effects of predation on multiple defensive traits [8]. Plastic changes in a predator's behaviour and life history and in predator-prey interactions may yield multiple defences [8]–[12]. Multipredator environments may also cause the evolution of multiple defence traits because prey species are exposed to a variety of predatory strategies that differ in their search and capture characteristics [13], [14]. Similarly, different defensive traits may be effective against the different attack strategies that the same predator can adopt, such as crush-searching [15] and shell entry-shell crushing [16], [17].

Another hypothesis states that defence by a prey species against the one attack strategy used by a single predator may result not from a single trait but from a combination of several traits [18], [19]. It is probable that prey species invest in multiple types of defensive traits because of the potential advantage of a combination of defences. Nonetheless, very few studies demonstrate that a single trait is insufficient to predict prey responses to a single predator and that a combination of different defensive traits is needed for optimal protection. In the present study, we test this hypothesis with a prey-predator system of two species of micro land snails.

Gastropod shells have been employed as a model to understand how anti-predator traits evolve because Gastropoda display a number of morphological traits (e.g., spines, thick shells, thick-lipped apertures) that serve to defend against predation. Furthermore, these traits are often plastic [20]–[28]. Although the defensive traits of land snails are not as obvious as those of marine snails, several examples of such traits involving shell shape and colour have been found [29]. The twisted shell of a land snail occurring on limestone outcrops in Borneo facilitates escape from attack by predatory slugs [30], [31]. Modifications of the aperture shape of land snails help their escape from a malacophagous snake [32]. Even after ingestion by a predator, certain micro land snails can survive in the predator's digestive system by sealing the aperture of the shell with a calcareous epiphragm or operculum [33].

Land snails often exhibit a number of denticles, protruding plates and lamellae within the aperture [34]. In certain species, the last part of the whorl is elongated and detached from the previous whorls, and the aperture protrudes from the shell [35]. Additionally, certain taxa of micro land snails sometimes develop both denticles and the protruding aperture on their shell. These unique structures are frequently hypothesised to serve as barriers against such predators as beetles, flatworms, and malacophagous snails because these predators commonly insert own head or body into the shells through the aperture to attack the snail [36]–[38]. However, no studies have tested the effectiveness of these traits in defence against predators, even though these traits offer an excellent opportunity to test the effectiveness of combinations of multiple types of defensive traits against attack by a single predator. In the present study, we address this issue with predation experiments that use the micro land snail *B. plicidens* as the prey and the micro land snail *Indoennea bicolor* as the predator. We artificially altered the characteristics of the shell traits of the prey and tested the effectiveness of each trait and of the traits in combination.

## Methods

**Figure 1:**
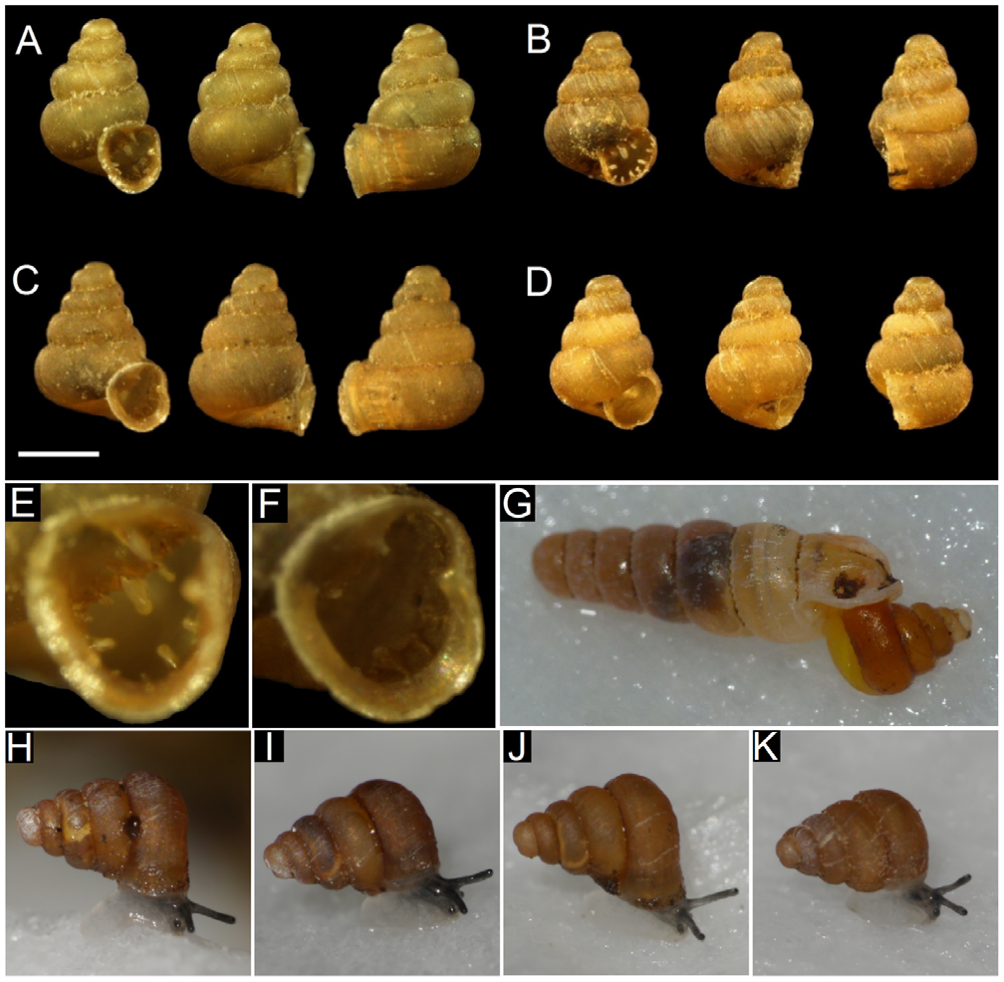
The images of shell state, predatory behaviour, and active state. (A) The shell of *Bensonella plicidens* in treatment *U*, (B) treatment *a*, (C) treatment *d*, and (D) treatment *a*+*d*. Scale bar  = 1 mm. (E) Detail of the denticles within the aperture of *B. plicidens* in treatment *U* and (F) treatment *d*. (G) Predatory behaviour of *Indoennea bicolor*. (H) The active state of *B. plicidens* in treatment *U*, (I) treatment *a*, (J) treatment *d*, and (K) treatment *a*+*d*.

*Bensonella plicidens* (Benson 1849) is a micro land snail (2 mm in diameter) with a protruded aperture containing 13–15 denticles (Figure 1E). These denticles occur at a position within the whorl tube approximately 400 μm from the surface of the aperture. The samples of *B. plicidens* used for the experiments were collected from two localities, Kanna (Gunma, Japan) (36° 07′ 13″N, 138° 55′ 13″E) and Kuma (Kumamoto, Japan) (32° 17′ 08″N, 130° 38′ 35″E), to reduce the impact on the individual populations of *B. plicidens*. In nature, a carnivorous snail *Sinoennea iwakawa* (Pilsbry 1900) has been known as a potentially sympatric predator of *B. plicidens*. However, in this study, we used *Indoennea bicolor* (Hutton 1834) as a substitute for this carnivorous snail because *S. iwakawa* is becoming a rare species in its natural habitats. Both of these small malacophagous species prey on snails by using their elongated body to enter the shell of the prey snail through the aperture (Figure 1G). *I. bicolor* were collected from Hateruma Island (Ryukyu, Japan) (24° 03′ 58″N, 123° 45′ 59″E). These snail species are not included on the red list of the International Union for Conservation of Nature (IUCN), and thus no specific permissions was required for this study.

In total, 140 individuals of *B. plicidens* were prepared with artificial alteration of the shell traits into four types (35 individuals each) for the experiments. In treatment *U*, the shells were unprocessed (Figure 1A, H). In treatment *a*, the protruded aperture of the shell was removed without damaging the denticles of the aperture and the soft body (Figure 1B, I). Because the denticles occur at a position in the tube deeper than this location, the removal of the protruded aperture did not damage the denticles In treatment *d*, all the denticles of the shells were removed, without damaging the aperture and the soft body (Figure 1C, F, J). For treatment *a*+*d*, both of treatments a and d were applied (Figure 1D, K). All the treatments were performed using a razor and sharp forceps under a stereoscopic microscope. To ascertain whether the treatments generated any unnecessary effect on the experiments, we examined the activity of the prey snails. Ten individuals of each of the four shell types were placed in a cage overnight under humid conditions (from 9 p.m. to 9 a.m.), and every 30 minutes we recorded whether they were moving (scored as 1) or not (scored as 0). The sum of the scores was regarded as the activity of each individual. There was no significant difference in the scores among the four shell types (χ^2^ = 0.359, *P* = 0.949), showing no effects of the treatment on the activity of the snails. In addition, there were no individuals that died within three days after treatment, suggesting that there were no effects of the treatment on the mortality of the prey except for predation because all the treatments were executed just prior to each experiment.

We prepared five small cages (30 mm×30 mm) under humid conditions. An altered *B. plicidens* and a starved *I. bicolor* were placed overnight (from 9 p.m. to 9 a.m.) in every cage. We confirmed that the cage width and period of the experiment were sufficient for predation by *I. bicolor* though preliminary experiments using another prey snail with a shell that has no denticles or protruded aperture. After placing a predatory snail and a prey snail in the same cage overnight, the condition of the prey snail was examined. If the shell of the prey snail was empty or the snail did not move again in the ensuing 24 hours, predation success was recorded. Each predator was then removed from the experiment for three days for starving. These scenarios were replicated seven times for every predator. Subsequent to the replications, the same experiments were conducted for the other three *B. plicidens* treatment without fixed order.

We performed chi-squared tests to examine the significance of the difference in the escape success among the prey treatments. The effects of the denticles and protrusion of aperture of the prey shell on the escape success from predator attack were then examined with a likelihood ratio chi-squared test using a binomial generalised linear mixed model (GLMM) with logit link. In the GLMM, the number of whole experimental replications for each predator was incorporated as a fixed effect, and the differences among the individual predators used in the experiments were treated as a random effect. The analyses were conducted with R 2.13.1 [39] and the lme4 package [40].

## Results

**Figure 2:**
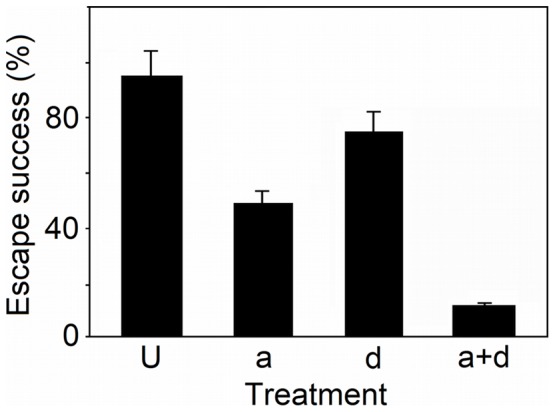
Bar graph of the results of predation experiments. The proportion of successful escapes (means across 35 trials using five predators +1 SE) of *Bensonella plicidens* from predation by *Indoennea bicolor* for each treatment.

Most of the *B. plicidens* individuals (94.3%) with unprocessed shells (treatment *U*) were able to escape from the attack by *I. bicolor*. However, the rate of successful escape decreased significantly when only the denticles were removed (treatment *d*) (χ^2^ = 12.3, *P*<0.001), with 74.3% of the snails surviving. When only the protruded aperture was removed (treatment *a*), the rate of successful escape also decreased significantly (χ^2^ = 30.8, *P*<0.001), and 48.6% of the snails survived (Figure 2). Thus, the loss of either of these shell traits caused a marked reduction in their defensive ability. If both of the denticles and the protruded aperture were removed (treatment *a*+*d*), only 11.5% of *B. plicidens* survived (Figure 2). A significant effect on the defence against predation was found for the denticles (χ^2^ = 11.1, *P*<0.001) and for the protruded aperture (χ^2^ = 23.8, *P*<0.001), though no difference in the predation success of each predator was detected between experimental replications (χ^2^ = 0.06, *P* = 0.8).

All of the prey snails that were attacked showed no shell damage other than that resulting from our artificial treatments. These results indicate that the denticles and protruded aperture function in defence against shell entry by predators and that the presence of denticles alone or the protruded aperture alone is not sufficient for defence against attack by a predator.

## Discussion

Defensive features on gastropod shells have been documented by a number of studies. These traits have been documented particularly often in species with induced polymorphisms. However, few studies have attempted to clarify the function features of the shell through its experimental manipulation. The present study experimentally documented that the denticles within the aperture and the protruded aperture of *B. plicidens* both individually function as a barrier to protect the soft body from attacks by a predator that enters the shell through the aperture. Each of these traits contributes to increase the defensive capability of the prey. However, the presence of only one of these traits is insufficient to protect the body of the prey from the attack of a predator via shell entry, and both traits are necessary for sufficient protection against attack via shell entry.

Micro land snails are targeted by many predators that can enter the shell through the aperture, e.g., terrestrial planarians [41], predatory gastropods [42] and the larvae of *Diptera* [43]. Although a malacophagous land snail was selected as the predator in our experiment, it is probable that the denticles within the aperture and the protruded aperture serve the same defensive function against other predators that attack snails by entering the shell. Gittenberger [37] suggests that the denticles in the aperture of *B. plicidens* might be effective as entanglements directed against minute arthropod antennae or legs. Although the hardness and shape of the tissue used to attack the victim differ among various predators, the basic methods used by these predators to enter the shell through the aperture are generally the same. The tactic necessary for successful predation involves a close approach to the soft parts of the prey, and the approach is performed with a long, narrow organ or with the extended body. Thus, increasing the length of the tube from the mouth of the shell to the position of the soft parts and creating barriers within the tube both serve to prevent the predator from approaching from outside the shell. However, the prey species faces a trade-off between defensive ability and other life history traits, and these defence traits are, therefore, costly. An overly narrow aperture and an excessively long tube may cause difficulties in feeding, mating and locomotion. One of the solutions to this problem is to increase the defensive ability as a whole by combining different defence traits, each of which does not increase the defensive ability substantially but is associated with a low cost. In addition, such combinations of different traits would be advantageous to prevent the attack of an enemy that specialises in a particular method of attack. Therefore, these combinations would also be advantageous in multipredator environments.

High predation pressure promotes the evolution of defensive traits, but the availability of resources, particularly calcium, constrains the development of defensive traits that are features of the shell [44]. It is, therefore, probable that strong defensive traits can evolve in those land snails that inhabit limestone outcrops in tropical regions, where predation pressure is the highest. In fact, land snails on limestone hills in tropical Asia and America frequently exhibit unique shell characters [30], [37], [45]–[47]. An extremely protruded aperture is found in certain species of Cyclophoridae and Diplommatinidae on limestone hills in southeastern Asia, and extremely developed denticles within the aperture are found in camaenid species on limestone outcrops in the tropical regions of America [35]. These observations suggest that these traits have evolved as an adaptation to defend against the high predation pressure in these regions. In addition to these traits, a number of unique potentially defensive traits (e.g., an expanded apertural lip, a meandering tube associated with the last whorl) develop in combination on the shell of these tropical snails. As in the case of *B. plicidens*, the combination of these characters would be highly effective at protecting the soft body. The present findings provide novel insight and lend importance to the combination of different traits to perform a particular function and the production of morphological diversity through prey-predator coevolution.
